# Patterns of multimorbidity and their association with edentulism: the moderating role of health literacy in the Lifelines Cohort

**DOI:** 10.1093/eurpub/ckag099

**Published:** 2026-06-17

**Authors:** Trishnika Chakraborty-Groot, Josue Almansa, Andrea F de Winter, Fernando Gregorio Linares-Jimenez, Annemarie A Schuller, Sijmen A Reijneveld

**Affiliations:** Department of Health Sciences, University Medical Center Groningen, University of Groningen, Groningen, The Netherlands; Department of Health Sciences, University Medical Center Groningen, University of Groningen, Groningen, The Netherlands; Department of Health Sciences, University Medical Center Groningen, University of Groningen, Groningen, The Netherlands; Department of Health Sciences, University Medical Center Groningen, University of Groningen, Groningen, The Netherlands; Center for Dentistry and Oral Hygiene, University Medical Center Groningen, Groningen, The Netherlands; TNO the Netherlands Organization for Applied Scientific Research, Leiden, The Netherlands; Department of Health Sciences, University Medical Center Groningen, University of Groningen, Groningen, The Netherlands

## Abstract

Multimorbidity may affect oral health, especially in individuals with limited health literacy (HL), or specific multimorbidity patterns; however, evidence remains scarce. Understanding these associations could improve early detection and prevention, both in clinical care and public health strategies. This study aimed to assess the association of multimorbidity and multimorbidity patterns with complete tooth loss, i.e. edentulism, and whether HL moderates this association. A total of 42 357 participants from the prospective multigenerational Dutch Lifelines Cohort Study were included. Multimorbidity was defined by ≥2 affected disease domains. Multimorbidity patterns were constructed with latent class analysis. Associations between multimorbidity (and patterns) with edentulism were assessed using logistic regression, with HL as a potential moderator. Models were adjusted for age, household income, and education. Edentulism prevalence was 9.5% and was higher among limited than adequate HL (14.5% vs. 7.9%) and among multimorbid vs. absent (19.5% vs. 7.7%). We identified five patterns: (1) Endocrinological + Psychiatry; (2) Cardiovascular + Endocrinological; (3) Neurological + Otorhinolaryngologic; (4) Endocrinological + Otorhinolaryngologic, and (5) Otorhinolaryngologic + Psychiatric. The odds of edentulism were 2.26 higher (95% CI: 2.06—2.47) in multimorbid. Pattern 2 (Cardiovascular + Endocrinological) had the highest odds ratio of edentulism (OR: 3.52, 95% CI: 3.03; 4.08) compared to multimorbidity absent. HL did not moderate the associations between multimorbidity and edentulism. Edentulism is more likely in the case of multimorbidity, especially if cardiovascular and endocrinological conditions co-occur, an explanation being the shared risk factors. This association remains the same across different HL levels. Our findings support collaborative medical and oral healthcare.

## Introduction

Oral diseases are a major global health issue, affecting approximately 3.5 billion individuals worldwide according to the WHO Global Oral Health Status Report (2022) [1]. Among these conditions, edentulism (complete tooth loss) poses a substantial challenge, with a prevalence of 7% for individuals aged 20 years or older, increasing to 23% among those aged 60 years or older [2]. These statistics underscore the urgent need to address oral health within the broader framework of noncommunicable disease prevention. Edentulism can be considered as the end-stage of oral problems, like periodontal disease, severe dental caries, and chronic systemic conditions. All of these share common risk factors such as smoking, unhealthy lifestyle choices, poor diet, and chronic inflammation [3]. Notably, these common risk factors are also associated with the development and progression of noncommunicable diseases and multimorbidity [4].

Multimorbidity, i.e. the co-occurrence of two or more chronic conditions in the same individual, impacts over half of individuals aged 60 years and older [5, 6]. Previous population-level cohort studies have already established bidirectional associations of oral diseases, such as periodontitis, with several cardiovascular diseases (stroke, myocardial infarction) [7], thyroid diseases (majorly, hypothyroidism) [8], and diabetes [9]. Emerging evidence indicates that *multimorbidity patterns* may be associated with oral diseases more significantly than single systemic conditions [4]. Along with existing evidence on shared risk factors like smoking and unhealthy diets, understanding multimorbidity patterns can help dentists and medical professionals better guide patients with multiple conditions towards preventing worsening of oral diseases and promoting oral health [10]. However, the evidence on the link between multimorbidity patterns and oral diseases remains scarce and has limitations. First, in previous studies, multimorbidity is often oversimplified, i.e. measured as a crude count [15] or binary [3, 11]. Second, majority studies were cross-sectional [3, 12] or used self-reported disease measurement [11–15]. This limits our understanding of causal pathways and disease-specific interactions with oral conditions.

Health literacy (HL) has been associated with both edentulism [16] and multimorbidity [17], raising the issue whether limited HL (LHL) may increase the likelihood of edentulism in the case of multimorbidity. HL is a critical social determinant of health and is defined as ‘the ability to access, understand, evaluate, and communicate information to manage health across the life course’ [21]. A recent meta-analysis reported indirect mechanisms, such as medication adherence, treatment burden, and patient-provider interactions, linking LHL to multimorbidity [17]. Despite its importance, evidence lacks on the role of HL in the relationship between multimorbidity and oral health. Therefore, this study aimed to explore the association of multimorbidity and multimorbidity patterns with edentulism and whether HL moderates this association, Fig. S1.

## Methods

### Study design and population

We utilized data from Lifelines, a large-scale population-based, multidisciplinary cohort of 167 729 individuals from the northern Netherlands, spanning three generations. Initially, participants were recruited through general practitioners, who further invited their family members. Lifelines employs a comprehensive set of investigations to assess biomedical, socio-demographic, behavioural, physical, and psychological factors influencing health and disease, with emphasis on multimorbidity and complex genetics [18].

This study included data for 152 894 adult participants, from which 94 698 were offered HL questionnaire at baseline follow-up between 2012 and 2015, and self-reported questionnaires about oral conditions (such as tooth loss, bleeding gums, etc) in the third assessment wave between 2019 and 2023. This resulted in 42 357 participants who completed the questionnaires on HL and oral conditions, and had a complete disease profile at baseline between 2007 and 2015.

The Lifelines Cohort Study follows the conventions outlined in the Declaration of Helsinki. The University Medical Center Groningen’s Medical Ethics Committee approved the Lifelines Cohort Study (approval number: METc 2007/152 and METc 2019/571), and all subjects provided written informed consent.

### Measures

#### Edentulism

Edentulism was assessed with three self-reported questions about the presence of teeth in the upper jaw and lower jaw, and the count of teeth in the mouth. The results were dichotomized as edentulism ‘absent’ or ‘present’. Edentulism ‘present’ refers to complete absence of teeth in upper and lower jaw. Cross-checking responses across these measures helped to reduce potential misclassification. Past studies have validated self-reported oral health conditions against clinical assessments, showing reasonable accuracy for certain measures, especially edentulism [19].

#### Single morbidities, DD, and multimorbidity

The presence of 38 morbidities was assessed through questionnaires, medication lists, clinical examinations, laboratory assessments, electrocardiography, and spirometry, and then classified according to the 11th edition of the International Statistical Classification of Diseases and Related Health Problems (ICD-11). Individuals needed to meet at least one diagnostic criterion to be classified as affected by a single morbidity [20, 21]. We then defined, ‘multimorbidity by disease count’, based on the count of confirmed diseases, as ‘present’ (≥2 disease count) vs. ‘absent’ (<2 disease count).

Subsequently, single morbidities were clustered into 10 mutually exclusive disease domains (DDs). This method of operationalization was in line with previous studies from Lifelines [20, 21]:

1. Gastrointestinal (GI),
2. Cardiovascular diseases (CVD),
3. Endocrinological (Endo),
4. Otorhinolaryngologic (ENT) and Respiratory,
5. Neurologic (Neuro),
6. Psychiatric (Psy),
7. Musculoskeletal disorders (MSD),
8. Coagulopathy and haematology (Hema),
9. Genitourinary (Uro), and
10. Dermatological (Derma).

Together, these 10 DDs represent overall health. We calculated ‘disease domain’ (DD) count as a composite endpoint, in which a DD (e.g. GI, CVD) was considered as ‘affected’, when at least one single morbidity was present within this DD shortly before and during the first visit at Lifelines outpatient clinic. ‘**Multimorbidity by disease domain**’ was subsequently dichotomized as ‘present’ when ≥2 DD affected vs. ‘absent’ (max 1 DD).

Cancer was excluded from these domains due to its varied physiopathology, risk factors, treatments, and prognoses across different types. In line with that, oncological conditions are directly related to the extraction of teeth with inflammatory foci to identify inflammations (focus screening). Hence, inclusion of cancer would reduce the precision of the multimorbidity classification. Based on Delphi’s consensus study, we excluded severe sensory conditions such as permanent visual impairment and permanent hearing disability that cannot be corrected and non-surveillance diseases, such as cataract [22].

#### Health literacy

HL was measured at baseline with the three validated self-report questions from Chew *et al.* [23]:

1. How often do you have trouble understanding your medical situation because you have difficulty with the written information?
2. How sure are you of yourself when you fill out medical forms?
3. How often does someone help you with reading the information materials from the hospital or another healthcare provider?

Participants responded to this questionnaire using a Likert-type scale (1–5). After reversing scores for the first and third question and summing all responses, a HL score of 3–15 was derived, dichotomized as ‘limited’ (3–12) vs. ‘adequate’ (13 or more). This cut-off reliably identified inadequate HL with strong sensitivity and specificity (ROC value of 0.74) [23].

#### Background variables

Background variables included age, gender, education, and household income. These were extracted from the baseline questions and categorized according to previous studies based on Lifelines [24]. Age at baseline assessments was dichotomized (≥65, <). Educational level was classified according to the International Standard Classification of Education into three categories: low, intermediate, and high [24]. Household income was classified according to the low-income threshold defined by Statistics Netherlands, into three categories: <1000 euros, 1000–2000 euros, and >2000 euros [25].

### Statistical analyses

The analyses were performed in four steps. First, we described the demographic and background characteristics of the included sample and the prevalence of edentulism within the categories of these variables.

Second, to construct multimorbidity patterns, latent class analysis (LCA) was performed on individuals with ‘multimorbidity by disease domain’ (i.e. ≥2 DD). This operationalization was preferred over ‘multimorbidity by disease count’ for three main reasons: (i) DD allows easier interpretation in terms of clinical significance and policy relevance, and ensures consistency with previous studies using Lifelines data [20, 21], (ii) mitigates the methodological limitations that several conditions had very low prevalence (*n* < 10), compromising statistical robustness, and (iii) to minimize the risk of misclassification inherent to self-reported data, conditions were grouped into broader DD rather than retained as individual diagnoses.

LCA is a model-based approach used to classify individuals into homogeneous subgroups within a heterogeneous population, where each class (multimorbidity pattern) is defined by a specific disease probability profile (i.e. the probability of endorsing a disease for individuals in that class). A series of LCA models ranging from 1 to 10 classes were estimated and compared based on (a) comparative goodness-of-fit indices such as the Bayesian information criterion (BIC), the Akaike information criterion (AIC), where lower values indicate better fit, (b) avoiding small class proportion and (c) theoretical *(clinical)* interpretability [26]. To ensure an optimal LCA solution, we used 250 random starting values. The substantive meaning of the classes was considered for the selection of the number of classes, i.e. whether the classes align with clinical knowledge. An *entropy index* value higher than 0.80 suggests highly discriminating latent classes, thus, negligible classification error when assigning individuals to their class with higher class-membership probability [27].

Third, we explored the association between multimorbidity (by disease count and DD) and edentulism using logistic regression (LR). Additionally, the association between multimorbidity patterns, constructed by LCA, and edentulism using LR. Missing values in covariates were at random.

Fourth, we examined whether HL moderates the above-mentioned associations between multimorbidity by DD and its patterns with edentulism. This was tested by including interaction terms (HL*Multimorbidity and HL*Multimorbidity patterns) in the LR models. All LR models were adjusted for age, household income, and education level.

As a sensitivity analysis, we repeated the moderation analysis of HL using ‘multimorbidity by disease count’. Now, a new multimorbidity category captured those with ≥2 diseases within single domain. This assessed the robustness of the moderation effect held across different multimorbidity definitions.

Data preparation and analyses were conducted using R version 3.6.2, and the LCA modelling was conducted using the poLCA package [28].

## Results

### Participant characteristics at baseline

Our total sample consisted of 42 357 participants (mean age 46 years, 59.5% female, and 15.4% multimorbidity present). There were no relevant differences that could lead to potential biases between the included and total sample, detailed in Table S1. Edentulism was approximately twice as prevalent in LHL vs. adequate HL (AHL) (14.5% vs. 7.9%) and highly prevalent in those with multimorbidity vs. without (19.5% vs. 7.7%), further see Table 1.

**Table 1.** Descriptives of the sample and prevalence of edentulism

	Study sample	Edentulism prevalence
Health literacy (HL)		
Limited (LHL)	10 194 (24.1%)	1480 (14.5%)
Adequate (AHL)	32 163 (75.9%)	2558 (7.9%)
Age		
18–64	39 818 (94.0%)	3109 (7.8%)
>64	2539 (6.0%)	929 (36.5%)
Household income (in EUR)		
<1000	4622 (12.6%)	501 (10.8%)
1000–2000	24 319 (66.4%)	2267 (9.3%)
>2000	7683 (21.0%)	607 (8.0%)
Gender		
Female	25 211 (59.5%)	2015 (8.0%)
Male	17 146 (40.5%)	2023 (11.8%)
Education level		
Low	511 (1.7%)	194 (37.9%)
Intermediate	16 709 (55.4%)	2185 (13.0%)
high	12 952 (42.9%)	1084 (8.4%)
Disease domain (DD) count		
0	22 002 (51.9%)	1292 (5.9%)
1	13 829 (32.6%)	1475 (10.6%)
2	4812 (11.4%)	840 (17.4%)
3	1312 (3.1%)	306 (23.3%)
≥4	402 (0.9%)	125 (31.1%)
Multimorbid by DD		
Present (≥2)	6526 (15.4%)	1271(19.5%)
Absent (<2)	35 831 (84.6%)	2767 (7.7%)
Total	42 357	4038 (9.5%)

### Identifying the latent class model

According to the goodness-of-fit indices, model fit continued to improve up to the 10-class models (Table S2). As goodness-of-fit did not yield a conclusive result, the final number of classes was determined primarily based on clinical interpretability. From five-class model onwards, we observed a stable ‘*Cardiovascular* + *Endocrinological*’ pattern, which appeared consistently with similar profiles and class sizes across models with higher numbers of classes. Other patterns, however, tended to split or merge combinations of the most prevalent domains (Neuro, ENT, Endo, CVD, and Psy) as the number of classes increased.

When comparing the five-class model to the six-class model, pattern 5 from the five-class model, representing 17.3% of sample (class size), defined by the ‘ENT (otorhinolaryngologic)’ domain (100% disease probability, in percentage) and the ‘psychiatric’ domain (43.8% disease probability), was split into two distinct patterns in the six-class model, as detailed in Table S3.

Although these two new classes appear to be more homogeneous than their original class, the association of these two new classes with edentulism were similar to that of their original class in the five-class solution. Furthermore, as the number of classes increased, classes became smaller, limiting the power to identify significant associations and modification. Thus, for parsimony and practicality, we continued our analyses with the **five-class solution.** We adopted class names based on the DD with the highest probabilities. The description of the patterns in the five-class model, Table S3 and Fi. 1, is as follows:

- Pattern 1 *(class size 21.6%),* with high disease probability Endo (72.2%) + Psy (45.5%).
- Pattern 2 *(class size 18.6%),* CVD (100%) + Endo (86.3%).
- Pattern 3 *(class size 26.0%),* Neuro (100%) + ENT and Respiratory (43.4%).
- Pattern 4 *(class size 16.5%)*, Endo (100%) + ENT and Respiratory (100%).
- Pattern 5 *(class size 17.3%),* ENT and Respiratory (100%) + Psy (43.8%).

Given that entropy was high, for the following analyses each individual was assigned to a class based on their posterior class-membership probability.

Within these five classes, we observed patterns that co-occur and share common underlying aetiologies and risk factors related to edentulism based on the literature. For instance, cardiovascular and endocrinological diseases share multiple risk factors, such as plasma lipid levels, blood pressure, glycaemic control, weight, and smoking status [29] and both these DDs, cardiovascular [30] and endocrinological [29] are associated with edentulism. Similarly, endocrinological diseases (such as diabetes, hypothyroidism) are associated with gene mutations with ENT manifestations because of mutations of genes encoding insulin and the GH receptor [31], and are associated with severe periodontal diseases [32].

### Association of multimorbidity (by disease count and by DD), and multimorbidity patterns with edentulism

The odds of edentulism were 2.12 higher (95% confidence interval (CI): 1.90; 2.37) in multimorbid by disease count compared to the non-multimorbid individuals, see Table 2. This result was similar with alternative definition of multimorbidity by DD. Regarding the multimorbidity patterns, Pattern 2 (Endo + CVD) showed the strongest association (odds ratio, OR = 3.52 (3.03; 4.08)) with edentulism compared to multimorbidity absent. We also examined each DD separately and found results consistent with the multimorbidity patterns: the endocrinological and cardiovascular domains showed the strongest individual associations with edentulism, Table S4.

**Table 2.** Prevalence rates of edentulism and the odds of edentulism for (A) multimorbidity by diseases count and (B) multimorbidity by disease domains (DDs) and its patterns, compared to multimorbidity absent

		Prevalence edentulism%	Odds ratio (OR)	OR (adj)^a^
A. Multimorbidity by disease count (≥2 diseases count)^b^	*Absent*	7.4	1 (ref)	1
	Present	18.2	2.68 (2.46; 2.92)	2.12 (1.90; 2.37)
B. Multimorbidity by disease domain (≥2 DD)^c^	*Absent*	7.7	1 (ref)	1
	Present	19.5	2.89 (2.69; 3.11)	2.26 (2.06; 2.47)
	Present, per pattern	7.7	1 (ref)	
	1 (Endo + Psy)^d^	18.1	2.61 (2.28; 3.00)	2.11 (1.77; 2.51)
	2 (CVD + Endo)^d^	32.7	5.85 (5.22; 6.56)	3.52 (3.03; 4.08)
	3 (Neuro + ENT)^d^	16.2	2.30 (2.04; 2.58)	1.76 (1.53; 2.04)
	4 (Endo + ENT)^d^	19.3	2.88 (2.50; 3.31)	1.98 (1.65; 2.37)
	5 (ENT + Psy)^d^	14.2	1.89 (1.60; 2.23)	1.75 (1.42; 2.15)

a Adjusted for age, household income, and education level.

b Reference category: multimorbidity absent (<2 disease count).

c Reference category: multimorbidity absent (<2 disease domain).

d Pattern 1—Endo + Psy: Endocrinological + Psychiatric diseases; Pattern 2—CVD + Endo: Cardiovascular + Endocrinological diseases; Pattern 3—Neuro + ENT: Neurological + Otorhinolaryngologic & Respiratory diseases; Pattern 4—Endo + ENT: Endocrinological + Otorhinolaryngologic & Respiratory diseases, and Pattern 5—ENT + Psy: Otorhinolaryngologic & Respiratory + Psychiatric diseases.

### Moderation effect of HL on the association between multimorbidity and multimorbidity patterns with edentulism

The association between multimorbidity by DD and edentulism was similar for individuals with LHL compared to those with AHL (OR: 1.07, 95% CI: 0.89–1.30), Table 3. HL did not moderate the associations between multimorbidity (and patterns) with edentulism.

**Table 3.** Moderation effects of health literacy (HL) on the associations of multimorbidity by disease domain (and its patterns) with edentulism

	Odds ratio; OR (confidence interval; CI)	OR^a^ (CI)
Overall multimorbidity: main effects and interaction		
Multimorbidity present (≥2 disease domain) vs. absent	2.81 (2.49; 3.18)	2.00 (1.78; 2.25)
Limited vs. adequate health literacy (HL)	1.86 (1.71; 2.02)	1.69 (1.53; 1.87)
Multimorbidity present × limited HL	1.02 (0.87; 1.18)	1.07 (0.89; 1.30)
Multimorbidity patterns: main effects		
Multimorbidity absent	1 (Ref)	
Pattern 1 (Endo + Psy)^b^	2.17 (1.76 ; 2.67)	1.83 (1.41; 2.38)
Pattern 2 (CVD + Endo)^b^	6.16 (5.27; 7.20)	3.76 (3.06; 4.63)
Pattern 3 (Neuro + ENT)^b^	2.06 (1.75; 2.42)	1.48 (1.20; 1.81)
Pattern 4 (Endo + ENT)^b^	2.66 (2.19 ; 3.24)	1.78 (1.38; 2.30)
Pattern 5 (ENT + Psy)^b^	1.86 (1.47; 2.34)	1.72 (1.28; 2.30)
Limited vs. adequate HL	1.86 (1.71; 2.02)	1.69 (1.53; 1.87)
Multimorbidity patterns: interaction		
Pattern 1 × limited HL	1.20 (0.87; 1.64)	1.21(0.82; 1.78)
Pattern 2 × limited HL	0.71 (0.55; 0.93)	0.74(0.53; 1.03)
Pattern 3 × limited HL	1.19 (0.91; 1.55)	1.24(0.89; 1.73)
Pattern 4 × limited HL	1.08 (0.78; 1.50)	1.20(0.80; 1.80)
Pattern 5 × limited HL	1.07 (0.74; 1.54)	1.08 (0.69; 1.69)

Reference: edentulism absent (presence of teeth), adequate health literacy (HL), and multimorbidity absent (<2 disease domain).

a Adjusted for age, household income, and education.

b Pattern 1—Endo + Psy: Endocrinological + Psychiatry; Pattern 2—CVD + Endo: Cardiovascular + Endocrinological; Pattern 3—Neuro + ENT: Neurological + Otorhinolaryngologic & Respiratory; Pattern 4—Endo + ENT: Endocrinological + Otorhinolaryngologic & Respiratory, and Pattern 5—ENT + Psy: Otorhinolaryngologic & Respiratory + Psychiatric.

### Sensitivity analysis

Consistent with the main analyses of multimorbidity by DD, HL did not significantly moderate the association between disease count (≥2 diseases) and edentulism, see Table S5.

## Discussion

This study demonstrated an association between multimorbidity and edentulism (complete tooth loss). The multimorbidity pattern characterized by endocrinological disorders and cardiovascular diseases had the strongest association with edentulism. HL did not moderate the association between multimorbidity (its patterns) and edentulism. Notably, this is the first study to examine, at the population level, the association between multimorbidity and its patterns with edentulism, and the moderating role of HL.

In this study, multimorbidity had a strong association with edentulism, consistent with previous cross-sectional studies, using self-reported measures for morbidity [4, 11, 12]. This association could be due to shared risk factors that contribute to the onset and development of both multimorbidity and oral diseases. These shared risk factors include smoking [29], poor diet, high sugar consumption [33], sedentary lifestyle [34], and psychosocial factors [35] and access to, and utilization of preventive health care [15] may further exacerbate this association, reinforcing health disparities.

Multimorbidity pattern characterized by endocrinological (such as hypercholesterolaemia, diabetes, and hypothyroidism) and cardiovascular diseases (such as coronary artery disease, heart failure, and atrial fibrillation) reported the strongest association with edentulism, aligning with past biological studies [9, 29, 36, 37]. Principal mechanisms linking chronic endocrinological and CVD diseases with oral diseases include: (i) spread of infection from the oral cavity due to transient bacteraemia, where oral microorganisms (such as *Porphyromonas gingivalis*) or their products enter the bloodstream and affect cardiac tissues, leading to coronary heart diseases [37]; (ii) induction of low-grade inflammation (LGI) arising from periodontal inflammation is strongly linked to the development and progression of chronic systemic diseases [36], and (iii) shared risk factors [13, 29], as discussed earlier. Therefore, the association between multimorbidity and edentulism may arise from shared social and behavioural risk, interacting biological pathways, or the progressive accumulation of risks over time, as evidenced by biomarkers.

**Figure 1.**
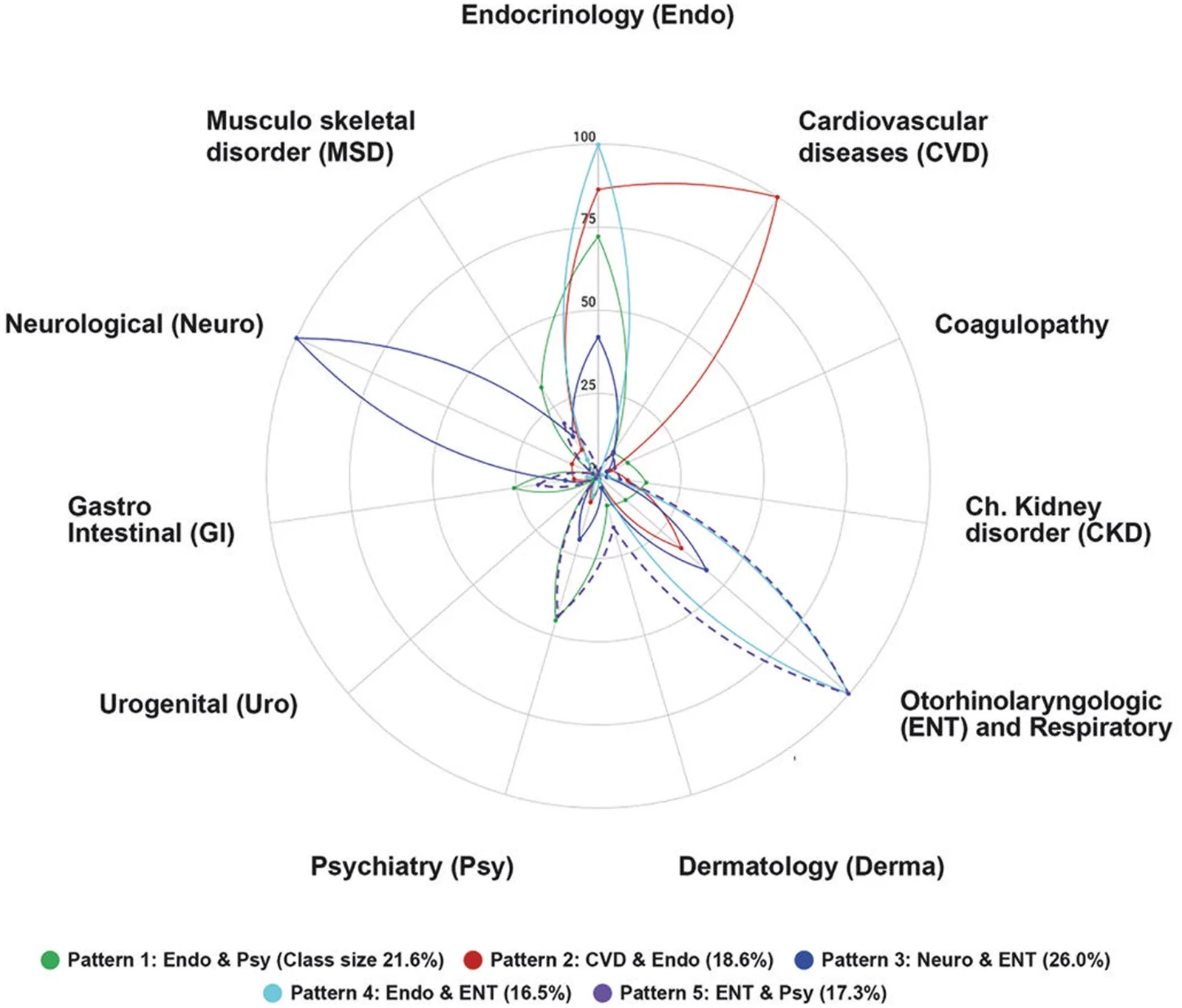
Multimorbidity patterns in the five-class multimorbidity model.

In this well-powered study, HL did not moderate the association between multimorbidity (or patterns) and edentulism. An explanation could be that HL functions only as a determinant of both multimorbidity and edentulism, i.e. that it is a common underlying cause and has an effect on oral health that is independent from the comorbidities/chronic conditions. HL may affect behavioural pathways that affect both general and oral health, consistent with the literature showing its role in edentulism [16] and multimorbidity [17, 24, 38]. However, HL may have a limited impact on certain multimorbidity patterns, particularly for conditions less reliant on individuals’ active use of health information, such as degenerative or hereditary diseases like osteoarthritis [38]. LHL could be a potential contributor to both multimorbidity and edentulism, but it does not have an additional impact once these outcomes have occurred.

### Strengths and limitations of the study

The strengths of this study are the use of a population-based sample, a large sample size with a wide selection of physical and mental chronic diseases, diagnosed by a combination of subjective and objective methods, using an internationally accepted coding system. Using LCA, a person-oriented rather than disease-oriented approach, aligns with current guidelines for patient-centred research and care [39].

Some limitations also warrant consideration. First, the moment of incidence of edentulism and chronic diseases remains uncertain, limiting causal inference. Although chronic conditions (2007–2015) and edentulism (2019–2023) were measured at different time points, this sequence ensured that exposure preceded outcome, supporting the proposed causal direction. Additionally, multimorbidity patterns in Lifelines have been reported to remain stable over time, suggesting disease classification was unlikely to change substantially [40]. Second, HL was measured with a focus on functional HL, which may have led to an underestimation of the full effects of low HL, such as communicative and critical literacy. While communicative HL enables individuals to engage in healthcare interactions, extract relevant information, and apply it to changing circumstances, critical HL refers to critically analyse information and making informed decisions. By excluding these dimensions, the measurement may not fully capture the complexities of how HL influences health behaviours and outcomes. Third, smoking was not included as confounder despite being a recognized risk factor for poor oral health, due to potential underreporting of smoking habits [16]. This may have introduced residual confounding and slight overestimation of associations.

Fourth, the exclusion of cancer from multimorbidity operationalization may have led to underestimation of the true burden of multimorbidity among low educated and low-income groups, given cancer’s higher prevalence in these population. Fifth, the five-class model may still contain heterogeneous individuals regarding their multimorbidity, but this is unlikely to affect our main conclusions. For example, the moderating effect of HL has remained non-significant in all models we estimated, using different multimorbidity definitions. Thus, it is unlikely that increasing the number of classes would yield significant effects, considering that the individuals (from the same sample) would be clustered into smaller classes.

### Implications

This study demonstrated an association between multimorbidity and edentulism, strongest for the pattern characterized by endocrinological and cardiovascular diseases. These results highlight the need for collaborative medical and dental healthcare to better manage multimorbidity and maintain oral health.

Our findings provide a foundation to advance research into causal associations between specific multimorbidity patterns and edentulism and explore shared biological pathways. Future studies should also examine common risk factors (healthcare utilization, lifestyle) using a common risk factor approach. Longitudinal research, capturing life course trajectory of multimorbidity and oral diseases, is needed to untangle causal pathways further.

## Conclusion

A pronounced association was observed between multimorbidity (also patterns) and edentulism. A primary focus should be on the multimorbidity pattern of cardiovascular and endocrinological diseases. HL had no moderating role in the association between multimorbidity and edentulism. A collaborative effort between dental and medical healthcare professionals is urgently needed to manage multimorbidity and reduce the associated burden of oral diseases.

## Supplementary Material

ckag099_Supplementary_Data

## Data Availability

Access to and use of Lifelines Cohort Study data and biosamples can be obtained via an electronic application portal, https://data-catalogue.lifelines.nl/.
