# Supplementary material for: Patterns of multimorbidity and their association with edentulism: the moderating role of health literacy in the Lifelines Cohort
Source: Eur J Public Health. 2026 Jun 17;36(4):ckag099. doi: 10.1093/eurpub/ckag099 (PMC13275122; doi:10.1093/eurpub/ckag099)
Supplement: ckag099_Supplementary_Data [file ckag099_supplementary_data.zip › ejph-2025-11-om-0994-File010.docx]

*Table S4*. Prevalence rates of edentulism and odds ratios (OR) of edentulism for each disease domain

| **Disease Domains** |  | Case/population | Prevalence edentulism (%) | OR | OR (adj)^1^ |
| --- | --- | --- | --- | --- | --- |
| **Endocrinology** | *absent* | *2632 / 34681* | 7.6 |  |  |
|  | present | 1406 / 7636 | 18.3 | 2.73 (2.55-2.93) | 2.10 (1.93-2.30) |
| **CVD** | *absent* | *3539 / 40564* | 8.7 |  |  |
|  | present | 499 / 1793 | 27.8 | 4.03 (3.62-4.50) | 2.77 (2.41-3.19) |
| **Coagulopathy** | *absent* | *3958 / 41546* | 9.5 |  |  |
|  | present | 58 / 485 | 12.0 | 1.29 (0.97-1.69) | 1.22 (0.87-1.23) |
| **CKD** | *absent* | *3910 / 41571* | 9.4 |  |  |
|  | present | 110 / 563 | 19.5 | 2.34 (1.89-2.88) | 1.70 (1.30-2.24) |
| **ENT and Respiratory** | *absent* | *2932 / 34358* | 8.5 |  |  |
|  | present | 1106 / 7999 | 13.8 | 1.72 (1.60-1.85) | 1.54 (1.40-1.68) |
| **Derma** | *absent* | *3960 / 41456* | 9.6 |  |  |
|  | present | 78 / 901 | 8.7 | 0.90 (0.70-1.13) | 0.86 (0.64-1.16) |
| **Psychiatry** | *absent* | *3706 / 39548* | 9.4 |  |  |
|  | present | 332 / 2809 | 11.8 | 1.30 (1.15-1.46) | 1.46 (1.26-1.68) |
| **GI** | *absent* | *3852 / 41076* | 9.4 |  |  |
|  | present | 186 / 1281 | 14.5 | 1.64 (1.40-1.92) | 1.62 (1.33-1.97) |
| **Neuro** | *absent* | *3516 / 38417* | 9.2 |  |  |
|  | present | 522 / 3940 | 13.2 | 1.52 (1.37-1.67) | 1.40 (1.24-1.59) |
| **Urogenital** | *absent* | *4020 / 42231* | 9.5 |  |  |
|  | present | 15 / 114 | 13.2 | 1.44 (0.80-2.40) | 1.10 (0.55-2.21) |
| **MSD** | *absent* | *3741 / 40825* | 9.2 |  |  |
|  | present | 297 / 1532 | 19.4 | 2.38 (2.09-2.71) | 1.80 (1.53-2.13) |

^1^ *Adjusted for age, household income and education level*
