# Supplementary material for: Patterns of multimorbidity and their association with edentulism: the moderating role of health literacy in the Lifelines Cohort
Source: Eur J Public Health. 2026 Jun 17;36(4):ckag099. doi: 10.1093/eurpub/ckag099 (PMC13275122; doi:10.1093/eurpub/ckag099)
Supplement: ckag099_Supplementary_Data [file ckag099_supplementary_data.zip › ejph-2025-11-om-0994-File008.docx]

| **MM models (number of classes)** | **AIC** | **BIC** | **Class size %** | | | | | | | | | | **Entropy** |
| --- | --- | --- | --- | --- | --- | --- | --- | --- | --- | --- | --- | --- | --- |
|  |  |  | **Class 1** | **Class 2** | **Class 3** | **Class 4** | **Class 5** | **Class 6** | **Class 7** | **Class 8** | **Class 9** | **Class 10** |  |
| 1 | 236237.7 | 236327.3 | 100.0 |  |  |  |  |  |  |  |  |  |  |
| 2 | 230052.8 | 230240.1 | 40.9 | 59.1 |  |  |  |  |  |  |  |  | 0.999 |
| 3 | 228024.7 | 228309.8 | 42.9 | 39.7 | 17.4 |  |  |  |  |  |  |  | 0.782 |
| 4 | 225750.9 | 226133.7 | 24.6 | 27.6 | 16.9 | 31.0 |  |  |  |  |  |  | 0.825 |
| **5** | **223859.4** | **224339.9** | **21.6** | **18.6** | **26.0** | **16.5** | **17.3** |  |  |  |  |  | **0.895** |
| 6 | 222392.4 | 222970.6 | 16.4 | 24.7 | 21.5 | 11.5 | 7.3 | 18.5 |  |  |  |  | 0.893 |
| 7 | 221167.2 | 221843.1 | 12.1 | 15.3 | 22.1 | 10.6 | 16.2 | 6.9 | 16.7 |  |  |  | 0.885 |
| 8 | 220054.2 | 220827.9 | 14.5 | 7.8 | 9.8 | 14.1 | 7.8 | 9.0 | 20.9 | 16.0 |  |  | 0.952 |
| 9 | 218579.3 | 219450.7 | 21.1 | 6.9 | 7.3 | 7.8 | 16.0 | 14.4 | 9.9 | 8.9 | 7.7 |  | 0.952 |
| 10 | 217472.1 | 218441.2 | 15.4 | 4.9 | 7.6 | 7.1 | 7.5 | 14.3 | 7.9 | 7.1 | 6.8 | 21.3 | 0.959 |

*Table S2*. Model fit indices of the LCA models (from 1 to 10 classes), for the estimation of multimorbidity patterns
