# Supplementary material for: Patterns of multimorbidity and their association with edentulism: the moderating role of health literacy in the Lifelines Cohort
Source: Eur J Public Health. 2026 Jun 17;36(4):ckag099. doi: 10.1093/eurpub/ckag099 (PMC13275122; doi:10.1093/eurpub/ckag099)
Supplement: ckag099_Supplementary_Data [file ckag099_supplementary_data.zip › ejph-2025-11-om-0994-File007.docx]

*Table S1.* Demographics of the included and excluded parts of the total Lifelines sample

|  | Included (N=42357) | Excluded (N=110537) | Total (N=152894) | Effect Size*^1^* |
| --- | --- | --- | --- | --- |
| Gender |  |  |  | 0.01 |
| Female | 25211 (59.5%) | 64209 (58.1%) | 89420 (58.5%) |  |
| Male | 17146 (40.5%) | 46294 (41.9%) | 63440 (41.5%) |  |
|  |  |  |  |  |
| Literacy level |  |  |  | 0.07 |
| limited | 10194 (24.1%) | 15831 (30.2%) | 26025 (27.5%) |  |
| adequate | 32163 (75.9%) | 36510 (69.8%) | 68673 (72.5%) |  |
|  |  |  |  |  |
| Multimorbidity |  |  |  | 0.02 |
| absent | 35831 (84.6%) | 91625 (82.9%) | 127456 (83.4%) |  |
| present | 6526 (15.4%) | 18911 (17.1%) | 25437 (16.6%) |  |
|  |  |  |  |  |
| Age |  |  |  | 0.05 |
| 18 – 64 | 39818 (94.0%) | 99697 (90.7%) | 139515 (91.6%) |  |
| 64+ | 2539 (6.0%) | 10222 (9.3%) | 12761 (8.4%) |  |
|  |  |  |  |  |
| Age at baseline |  |  |  |  |
| Mean (SD) | 46.663 (11.5) | 43.999 (13.6) | 44.731 (13.1) |  |
| Median (Q1, Q3) | 47.000 (40.0, 54.0) | 43.000 (34.0, 51.0) | 45.00 (36.0, 52.0) |  |
|  |  |  |  |  |
| Equivalized Household income | |  |  |  |
| Mean (SD) | 1628.389 (572.348) | 1488.791 (576.473) | 1529.844 (578.767) |  |
| Median (Q1, Q3) | 1590.990  (1237.437, 1944.544) | 1453.444  (1125.000, 1875.000) | 1587.713  (1125.000, 1875.000) |  |
|  |  |  |  |  |
| Education level |  |  |  | 0.06 |
| low | 511 (1.7%) | 3829 (4.5%) | 4340 (3.8%) |  |
| intermediate | 16709 (55.4%) | 46211 (54.1%) | 62920 (54.5%) |  |
| high | 12952 (42.9%) | 35328 (41.4%) | 48280 (41.8%) |  |
|  |  |  |  |  |
| Edentulism |  |  |  | 0.01 |
| Present | 4038 (9.5%) | 938 (9.0%) | 4976 (9.4%) |  |
| Absent | 38319 (90.5%) | 9449 (91.0%) | 47768 (90.6%) |  |
|  |  |  |  |  |
| Disease domain present score | |  |  | 0.03 |
| 0 | 22002 (51.9%) | 56273 (50.9%) | 78275 (51.2%) |  |
| 1 | 13829 (32.6%) | 35352 (32.0%) | 49181 (32.2%) |  |
| 2 | 4812 (11.4%) | 13266 (12.0%) | 18078 (11.8%) |  |
| 3 | 1312 (3.1%) | 4131 (3.7%) | 5443 (3.6%) |  |
| >=4 | 402 (0.9%) | 1514 (1.4%) | 1916 (1.3%) |  |
| Disease count present score | |  |  | 0.03 |
| 0 | 22002 (51.9%) | 56274 (50.9%) | 78276 (51.2%) |  |
| 1 | 12185 (28.8%) | 30806 (27.9%) | 42991 (28.1%) |  |
| 2 | 5213 (12.%) | 13895 (12.6%) | 19108 (12.5%) |  |
| 3 | 1882 (4.4%) | 5626 (5.1%) | 7508 (4.9%) |  |
| >=4 | 1075 (2.5%) | 3936 (3.6%) | 5011 (3.3%) |  |

*^1^Effect sizes are expressed as difference between included and excluded part. For continuous variables (baseline age, household income) Cohen's d was calculated. For categorical variables (e.g., gender, literacy level), Cohen’s omega was calculated. The number of participants are shown for categorical variables. Means, standard deviations, the median and interquartile range are given for continuous variables.*
