# Supplementary material for: Patterns of multimorbidity and their association with edentulism: the moderating role of health literacy in the Lifelines Cohort
Source: Eur J Public Health. 2026 Jun 17;36(4):ckag099. doi: 10.1093/eurpub/ckag099 (PMC13275122; doi:10.1093/eurpub/ckag099)
Supplement: ckag099_Supplementary_Data [file ckag099_supplementary_data.zip › ejph-2025-11-om-0994-File009.docx]

*Table S3*. Disease probability profiles (in percentages) of disease domains for the five and six class models.

1. Five- class model

| **Disease domains** | Pattern 1  (21.6%) | Pattern 2 (18.6%) | Pattern 3 (26.0%) | Pattern 4 (16.5%) | Pattern 5  (17.3%) |
| --- | --- | --- | --- | --- | --- |
| Endocrinology (Endo) | **72.2** | **86.3** | 41.7 | **100** | 0.3 |
| Cardiovascular diseases (CVD) | 8.3 | **100** | 8.5 | 0.1 | 8.0 |
| Coagulopathy (Hema) | 9.7 | 3.8 | 2.8 | 1.0 | 5.3 |
| Ch. Kidney disorder (CKD) | 14.5 | 9.1 | 2.7 | 3.3 | 6.8 |
| Otorhinolaryngologic (ENT) and Respiratory | 10.8 | 33.3 | **43.4** | **100** | **100** |
| Dermatology (Derma) | 9.3 | 1.0 | 3.6 | 1.5 | 16.3 |
| Psychiatry (Psy) | **45.5** | 8.2 | 19.8 | 6.4 | **43.8** |
| Urogenital (Uro) | 2.2 | 0.6 | 0.9 | 0.3 | 1.0 |
| Gastro Intestinal (GI) | 25.7 | 7.3 | 10.1 | 3.4 | 18.3 |
| Neurological (Neuro) | 1.1 | 8.7 | **100** | 0.0 | 1.7 |
| Musculo skeletal disorder (MSD) | 31.8 | 9.4 | 14.3 | 6.0 | 18.9 |

1. Six-class model

| **Disease domains** | Pattern1 (16.4%) | Pattern 2 (24.7%) | Pattern 3 (21.5%) | Pattern 4 (11.5%) | Pattern 5  (7.3%) | Pattern 6  (16.5%) |
| --- | --- | --- | --- | --- | --- | --- |
| Endocrinology (Endo) | 100 | 44.8 | 72.3 | 1.0 | 0.3 | **85.8** |
| Cardiovascular diseases (CVD) | 0.0 | 9.3 | 8.3 | 9.5 | 3.4 | **100** |
| Coagulopathy (Hema) | 0.9 | 2.9 | 9.9 | 7.0 | 1.9 | 3.8 |
| Ch. Kidney disorder (CKD) | 3.2 | 2.8 | 14.6 | 10.1 | 0.6 | 9.1 |
| Otorhinolaryngologic (ENT) and Respiratory | 100 | 39.1 | 11.3 | **100** | **100** | 33.7 |
| Dermatology (Derma) | 1.4 | 3.2 | 9.4 | 23.7 | 3.4 | 1.0 |
| Psychiatry (Psy) | 6.3 | 21.5 | 45.8 | 0.5 | **100** | 8.4 |
| Urogenital (Uro) | 0.3 | 0.9 | 2.2 | 1.3 | 0.4 | 0.6 |
| Gastro Intestinal (GI) | 3.3 | 10.0 | 25.8 | 24.8 | 7.0 | 7.2 |
| Neurological (Neuro) | 0.0 | 100 | 0.0 | 16.8 | 0.1 | 8.1 |
| Musculo skeletal disorder (MSD) | 5.9 | 14.4 | 31.9 | 26.5 | 6.3 | 9.2 |
