# Supplementary material for: Patterns of multimorbidity and their association with edentulism: the moderating role of health literacy in the Lifelines Cohort
Source: Eur J Public Health. 2026 Jun 17;36(4):ckag099. doi: 10.1093/eurpub/ckag099 (PMC13275122; doi:10.1093/eurpub/ckag099)
Supplement: ckag099_Supplementary_Data [file ckag099_supplementary_data.zip › ejph-2025-11-om-0994-File011.docx]

*Table S5*. Sensitivity analyses using multimorbidity definition based by disease count (A) descriptives and (B) moderation effect of HL

1. Descriptives

|  | Edentulism  (N=4038) | Non- Edentulism  (N=38319) | Total (N=42357) |
| --- | --- | --- | --- |
| **Disease count** |  |  |  |
| 0 | 1292 (32.0%) | 20710 (54.0%) | 22002 (51.9%) |
| 1 | 1256 (31.1%) | 10929 (28.5%) | 12185 (28.8%) |
| 2 | 825 (20.4%) | 4388 (11.5%) | 5213 (12.3%) |
| 3 | 366 (9.1%) | 1516 (4.0%) | 1882 (4.4%) |
| ≥4 | 299 (7.4%) | 776 (2.0%) | 1075 (2.5%) |
| **Multimorbidity by disease count** | |  |  |
| Present  (>2 disease count) | 1490 (36.9%) | 6680 (17.4%) | 8170(19.3%) |

1. Moderation effect of HL

|  |  | OR*^1^*(adj) | CI*^1^* | |
| --- | --- | --- | --- | --- |
| **Multimorbidity absent (<2 disease count)** | | 1 (Ref) |  | |
| Multimorbidity domain patterns absent, but >2 diseases count | | 1.71 | | 1.35; 2.15 |
| **Multimorbidity patterns:** Main effects | Pattern 1 (Endo +Psy)^2^ | 1.87 | | 1.44 ; 2.43 |
|  | Pattern 2 (CVD+ Endo)^2^ | 3.88 | | 3.15 ; 4.78 |
|  | Pattern 3 (Neuro + ENT)^2^ | 1.52 | | 1.24 ; 1.87 |
|  | Pattern 4 (Endo +ENT)^2^ | 1.84 | | 1.43 ; 2.38 |
|  | Pattern 5 (ENT +Psy)^2^ | 1.74 | | 1.30 ; 2.34 |
| Limited vs. adequate HL |  | **1.68** | | **1.51; 1.86** |
| **Multimorbidity patterns:** Interaction | Multimorbidity domain patterns absent, but >2 diseases count | 0.70 | | 0.47; 1.03 |
|  | Pattern 1 X limited HL | 1.19 | | 0.81; 1.76 |
|  | *Pattern 2 X limited HL* | *0.72* | | *0.52; 1.01* |
|  | Pattern 3 X limited HL | 1.21 | | 0.87; 1.68 |
|  | Pattern 4 X limited HL | 1.17 | | 0.78; 1.76 |
|  | Pattern 5 X limited HL | 1.05 | | 0.67; 1.65 |

*^1^Adjusted for age, household income and education*

*^2^****Pattern1 Endo + Psy****: Endocrinological + Psychiatric diseases;*

***Pattern 2 CVD + Endo:*** *Cardiovascular + Endocrinological diseases;*

***Pattern 3 Neuro + ENT:*** *Neurological + Otorhinolaryngologic & Respiratory diseases;*

***Pattern 4 Endo + ENT:*** *Endocrinological + Otorhinolaryngologic & Respiratory diseases, and*

***Pattern 5 ENT + Psy:*** *Otorhinolaryngologic & Respiratory + Psychiatric diseases*
