# Supplementary figures and images for: Patterns of multimorbidity and their association with edentulism: the moderating role of health literacy in the Lifelines Cohort
Source: Eur J Public Health. 2026 Jun 17;36(4):ckag099. doi: 10.1093/eurpub/ckag099 (PMC13275122; doi:10.1093/eurpub/ckag099)

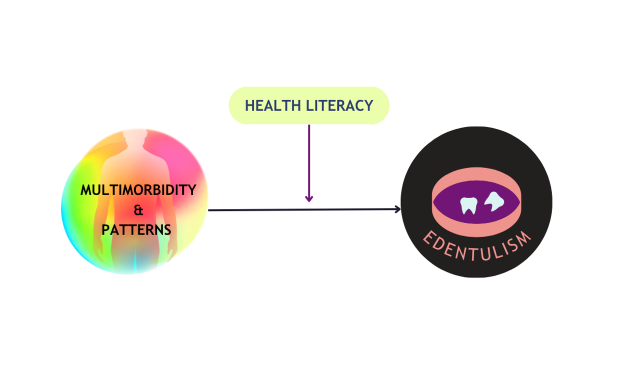

Supplement: ckag099_Supplementary_Data [file ckag099_supplementary_data.zip › ejph-2025-11-om-0994-File006.tif]
